# In Vivo Assessment of Benzoporphyrin Uptake and Singlet Oxygen Generation in Mice for Photodynamic Therapy Monitoring

**DOI:** 10.1021/acsphotonics.5c02685

**Published:** 2026-01-08

**Authors:** Vikas Vikas, Baozhu Lu, Weibing Yang, Brian C. Wilson, Timothy C. Zhu, Robert H. Hadfield

**Affiliations:** † James Watt School of Engineering, 3526 University of Glasgow, Glasgow G12 8LT, U.K.; ‡ Department of Radiation Oncology, 6572 University of Pennsylvania, Philadelphia, Pennsylvania 19104, United States; § Department of Medical Biophysics, 7989 University Health Network/University of Toronto, Toronto M5G 1L7, Canada

**Keywords:** singlet oxygen, benzoporphyrin derivative, laser medicine, photodynamic therapy, photon counting, dosimetry

## Abstract

The efficacy of photodynamic therapy (PDT) is strongly influenced by the biodistribution of the photosensitizer and the local generation of ^1^O_2_ within tumor tissue. However, real-time in vivo monitoring of these critical parameters for clinically approved photosensitizers remain a major challenge in translational photodynamic research. We report a novel portable bifurcated fiber-coupled time-resolved singlet oxygen luminescence detection instrument combining an integrating pulsed 690 nm diode laser system. Using this instrument, ^1^O_2_ signal is estimated corresponding to the uptake kinetics of the clinical photosensitizer benzoporphyrin derivative (BPD) in tumor-bearing mice up to 3 h post injection along with pre- and post-PDT ^1^O_2_ luminescence were measured in the same mice. The measured ^1^O_2_ counts correlate with the increase in BPD accumulation in the tumor region from 15 min to 2 h post injection and then remain constant in the 2–3 h measurement period. A strong positive correlation was observed between local BPD uptake and singlet oxygen signal. The estimated lifetime of ^1^O_2_ in vivo was 0.25–0.35 μs. The TSOLD system provided consistent, noninvasive readouts of ^1^O_2_ generation in real time with minimal background interference. Control experiments using BPD-free conditions confirmed the specificity of the detected signal. This study demonstrates a novel, noninvasive optical approach for simultaneous murine in vivo quantification of photosensitizer uptake and singlet oxygen production during PDT. This portable TSOLD instrument enables dynamic monitoring of therapeutic conditions in preclinical cancer models and has potential for future adaptation to clinical settings, supporting more precise and personalized PDT planning and dosimetry.

## Graphical abstract

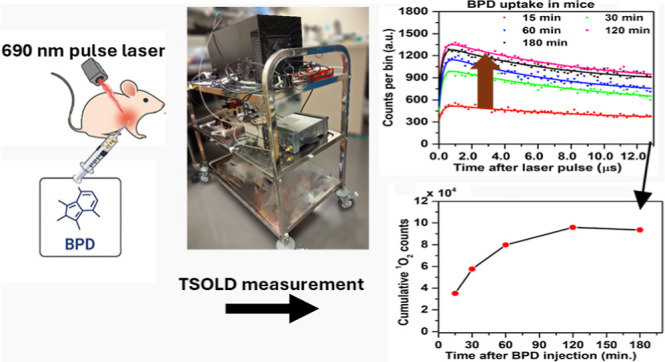

## 1 Introduction

Photodynamic therapy (PDT) is a minimally invasive cancer treatment that uses a photosensitizing agent, light of a specific wavelength to match the photosensitizer absorption spectrum, and molecular oxygen to induce cell damage in targeted tissues.^1,2^ Following light activation to an initial singlet excited state and intersystem crossing to a long-lived triplet state, the energy is transferred to adjacent oxygen molecules to produce singlet oxygen (^1^O_2_).^3,4^ This highly reactive species can trigger mitochondrial, lipid, DNA and vascular damage.^5–7^ Leading to tumor cell death by multiple pathways.^8,9^ This modality has proven effective across a range of malignancies, including head and neck cancers, nonsmall cell lung carcinoma (NSCLC) and esophageal cancer.^10–12^ Its selective action offers several advantages over conventional therapies, including reduced systemic toxicity, repeatability and the ability to preserve normal tissue architecture. However, the efficacy of PDT is directly dependent on both the local concentration of photosensitizer and the amount of ^1^O_2_ generated in the tumor microenvironment. Photosensitizer heterogeneity of uptake, oxygenation status and inhomogeneous optical properties of the tissue can lead to uneven therapeutic effects. Thus, there is a need for real-time, noninvasive methods that can monitor the biodistribution of photosensitizer and the generation of ^1^O_2_ in vivo to guide and customize PDT treatments.

Given the significance of ^1^O_2_ to the PDT effect, its real-time, in vivo quantification remains a relevant challenge. The detection of the ^1^O_2_ → ^3^O_2_ transition by its luminescence emission is the most direct approach.^13–15^ Some PDT dosimetry methods, such as multispectral singlet oxygen luminescence dosimetry (MSOLD), luminescence microspectroscopy, and continuous SOLD (CSOLD) have demonstrated real-time monitoring of singlet oxygen in different biological media with different photosensitizers and light fluence rates.^16–19^ However, due to the very short lifetime of ^1^O_2_ in tissue that results in the luminescence signal being very weak, detection of the ^1^O_2_ luminescence with a high signal-to-noise ratio (SNR) is still technically challenging. Time-resolved single-photon dosimetry (TSOLD) with gating allows subtraction of the photosensitizer and tissue fluorescence and phosphorescence that form a substantial background, allowing more robust measurements that are independent of the tissue conditions.^4,15,20–23^ The integration of high-sensitivity single-photon detectors, such as superconducting nanowire single-photon detectors (SNSPDs) and InGaAs single-photon avalanche diodes, has significantly improved the efficiency of ^1^O_2_ luminescence detection.^20,24–28^ However, there remains a need for a portable fiber probe-coupled TSOLD system that is suitable for preclinical and potential clinical use. The system reported here demonstrates significant potential for real-time detection of ^1^O_2_, particularly in achieving an improved SNR.

Liposomal benzoporphyrin derivative (BPD), also known as verteporfin, is a second-generation photosensitizer that is FDA-approved and has shown significant clinical utility.^29,30^ The near-infrared absorption and high singlet oxygen quantum yield of BPD allow for effective generation of cytotoxic reactive oxygen species with greater depth penetration, thereby permitting BPD to treat a wide variety of malignancies.^31^ Already FDA-approved for ocular disease, BPD is in clinical trials for solid tumors and metastatic lesions, exploiting its rapid clearance and good safety profile.^31,32^ Efficient BPD uptake by target cells is essential to achieve the maximum PDT efficacy, as the intracellular concentration dictates the spatial distribution and extent of cell killing.^33^ In addition, accurate ^1^O_2_ detection and quantification are significant in the case of BPD-mediated PDT, in which singlet oxygen is the primary cytotoxic agent.^34,35^

There is currently a gap in the instrumentation available for TSOLD, for use either in preclinical animal models or in patients. Here, we present a portable fiber-coupled TSOLD instrument using a 690 nm pulsed diode laser, which is suitable for in vivo detection of ^1^O_2_ generated by BPD. We demonstrate its use in radiation-induced fibrosarcoma (RIF) tumors in mice, both for singlet oxygen measurements and to estimate the BPD uptake in tumor pre and post PDT. By performing both assessments in the same tumors, we directly correlated photosensitizer accumulation with ^1^O_2_ generation efficiency under 690 nm excitation, providing a comprehensive evaluation of PDT photochemical dynamics in vivo. To our knowledge, this is the first instrument designed specifically for in vivo detection of ^1^O_2_ at 690 nm. The combination of fiber-based delivery and compact detection hardware enables minimally invasive measurements in live animals with high temporal resolution. This dual-parameter approach addresses current limitations in PDT dosimetry by linking photosensitizer pharmacokinetics to actual reactive oxygen species production under treatment conditions.

## 2 Materials and Methods

### 2.1 TSOLD Instrument for In Vivo Singlet Oxygen Detection

A custom-built portable Time-Resolved Singlet Oxygen Luminescence Detection (TSOLD) system was developed to quantify in vivo ^1^O_2_ generation following BPD administration, as shown in Figure 1. The system consists of a fiber-coupled diode laser with a pulse-current source, a combination of parabolic mirrors for coupling the incident beam and detection of ^1^O_2_ illumination through a bifurcated fiber (FCR-7UVIR200-2-ME-FC, Avantes, USA), a fiber-coupled single-photon avalanche diode (SPAD) detector, and time-tagger electronics for time-correlated single-photon counting (TCSPC).

**Figure 1:**
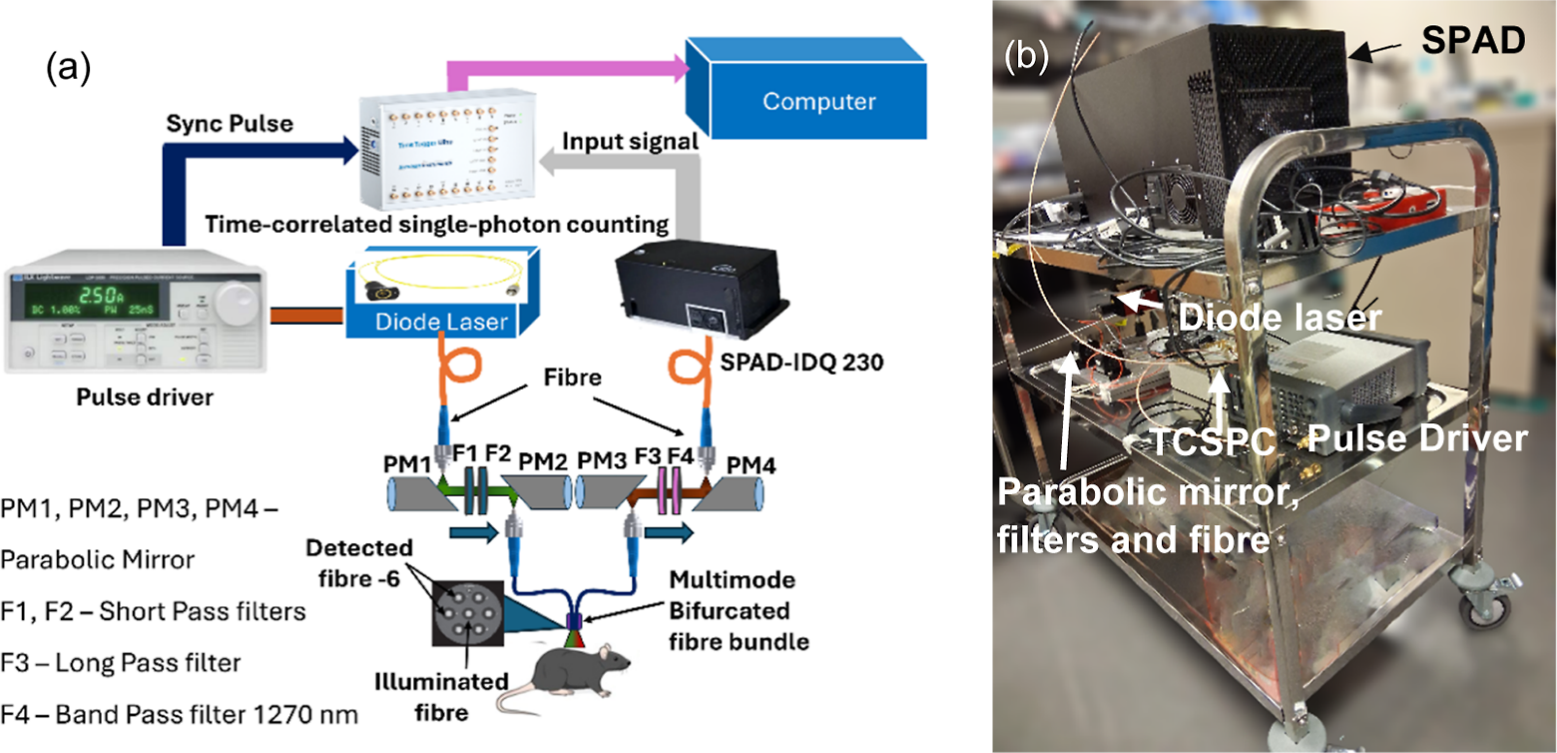
(a) Schematic and optical setup of the portable TSOLD instrument for in vivo ^1^O_2_ detection. An optical beam from an in-house fiber-coupled nanosecond pulse diode laser illuminates the tumor target through a bifurcated fiber bundle, parabolic mirrors, and short-pass filters. The collected 1270 nm luminescence emission is coupled into an InGaAs-SPAD through long- and band-pass filters, and a bifurcated fiber (central core for illumination delivery; 6 × outer cores for infrared luminescence collection and detection). The TCSPC then generates time histograms of the ^1^O_2_ luminescence signal. (b) Photograph of the portable TSOLD instrument.

A 690 nm pulsed diode laser was fabricated by using single mode fiber coupled pigtailed diode laser (LP685-SF15-250613-79, Thorlabs, Newton, NJ, USA) driven by a pulsed-current source (LDP-3830-120 V, MKS Newport Corporation, MA, USA). The wavelength of 690 nm was selected, as corresponding to the second highest absorption peak of BPD, to provide maximum penetration depth in tissue, as shown in Figure 2a. The measured absorption spectrum of the BPD and pulsed diode laser spectrum are shown in Figure 2, illustrating the matching of the output laser beam spectrum with the BPD absorption. In this present study, a laser beam with a pulse width of 130 ns, repetition rate of 78 kHz, and an average optical power of 2.3 mW was used. The beam from the pigtailed laser first passed through a 25 mm short-pass filter (FESH0950, Thorlabs, Newton, NJ, USA; optical density (OD) = 5.0 for wavelengths >950 nm) and a band-pass filter (FGS550, Thorlabs; transmission range: 304–785 nm, OD = 4.0) to suppress unwanted infrared and out-of-band light, and coupled into a bifurcated fiber bundle using a multimode in-line fiber filter mount (FOFMA/M, Thorlabs, Newton, NJ, USA). The output beam from the bifurcated fiber was used to illuminate the tumor with a spot size of 1–2 mm. The ^1^O_2_ luminescence emission at 1270 nm was collected through the same fiber bundle and transmitted to the second end of the bifurcation. This end was coupled to a second multimode in-line fiber filter mount, and the collected light then passed through a series of optical filters, including a long-pass filter (FELH1200, Thorlabs; OD = 5.0 for wavelengths <1200 nm) and a narrow band-pass filter (1270BP20, Omega Optical, Brattleboro, VT, USA; bandwidth: 1260–1280 nm, out-of-band OD = 6.0) to isolate the ^1^O_2_ luminescence. The filtered emission was subsequently coupled into a multimode optical fiber (core diameter: 65 μm) and directed to a single-photon avalanche diode (SPAD) detector (ID230, ID Quantique, Geneva, Switzerland) using a parabolic mirror for optimal coupling efficiency. The TCSPC module (Time Tagger Ultra, Swabian Instruments, Stuttgart, Germany) recorded photon arrival times by receiving a START signal from the pulse driver via an electrical synchronization pulse, while the detector output provided the STOP signal. This configuration enabled the construction of temporal histograms from single-photon detection events. To minimize timing errors due to pulse pile-up, which can distort the accurate estimation of ^1^O_2_ lifetime, the photon detection count rate was maintained below 5% of the excitation laser pulse repetition rate.

**Figure 2:**
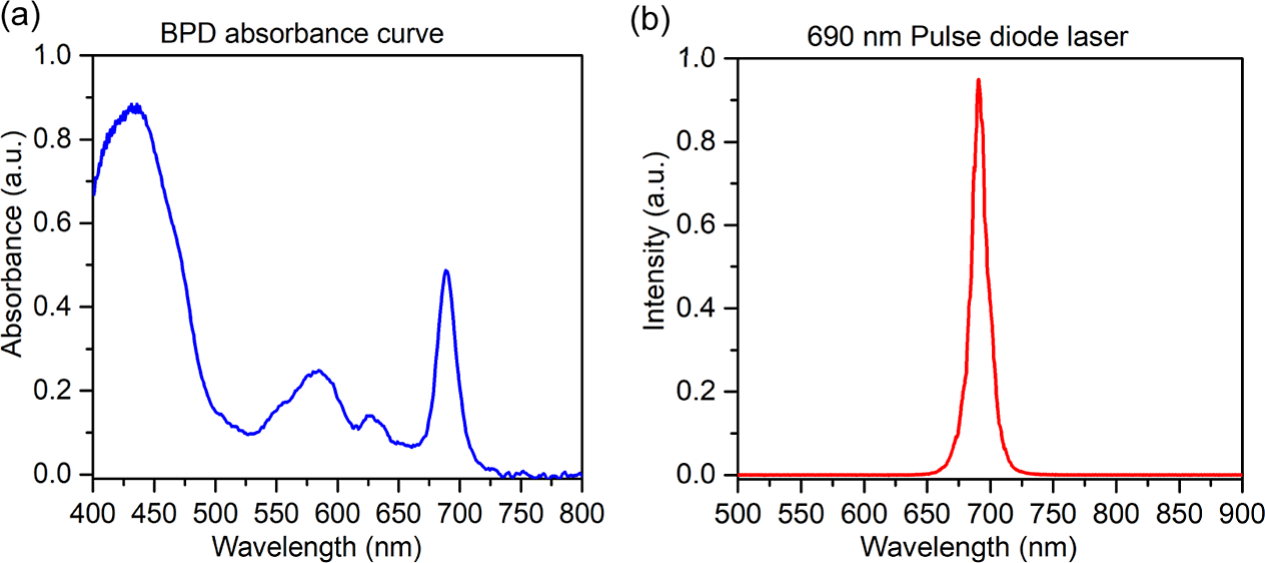
(a) Measured absorption spectrum of BPD in deionized water, showing the secondary absorbance peak at 690 nm. (b) Emission spectrum of the diode laser, with a peak at 690 nm.

### 2.2 Tumour Model

Radiation-induced fibrosarcoma (RIF) cells were cultured and injected into the right shoulder of 6–8 week old female C3H mice (Charles River Laboratories, Kingston, NY, USA) at a concentration of 1 × 10^7^ cells/mL in a 30 μL volume.^36,37^ Before cell injection, the fur over the injection site was clipped with a hair trimmer. To minimize background fluorescence interference from dietary chlorophyll, the mice were placed on a chlorophyll-free (alfalfa-free) diet (Harlan Laboratories, Indianapolis, IN, USA) immediately after cell injection. The development of the tumor was monitored, and mice were selected for treatment when tumors reached approximately 3–5 mm in diameter. Before treatment, the tumor area was depilated using a commercial hair removal cream (Nair, Church & Dwight Co., Inc., Ewing, NJ, USA). The FDA-approved benzoporphyrin derivative (Visudyne, QLT Ophthalmics, Inc., Canada) was injected via the mice’s tail vein at a concentration of 2 mg/kg. All procedures were approved by the Institutional Animal Care and Use Committee (IACUC) of the University of Pennsylvania, with animal care supervised by the U. Penn. Laboratory Animal Resources. During treatment, the mice were anesthetized by isoflurane and maintained at 37 °C on a temperature-controlled heating pad, as shown in Figure 3.

**Figure 3:**
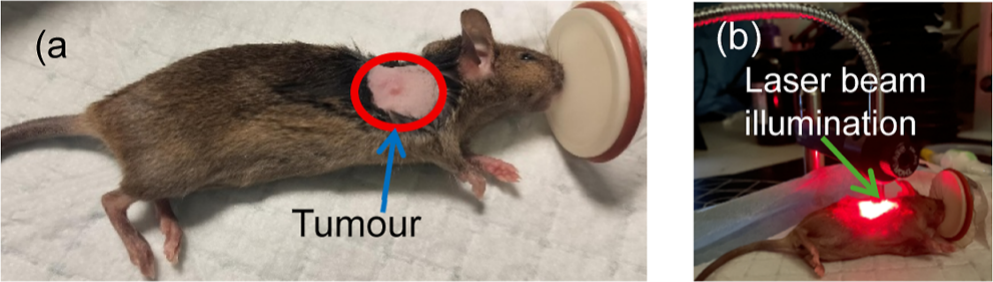
Photograph of tumor-bearing C3H mice (a) with visible subcutaneous RIF tumor (∼3–5 mm) prior to laser illumination, and (b) under 690 nm laser illumination at the tumor site, during TSOLD measurement.

### 2.3 Determination of Singlet Oxygen Lifetime

The lifetime of ^1^O_2_ plays an important role in determining the cell-killing efficiency. This was determined from the time-resolved histograms. A control decay curve, measured from nonphotosensitized tissue, was subtracted from the raw signal to eliminate background and autofluorescence contributions. The resulting curves were fitted using a biexponential function^25,26^

[O21]=A·τDτT−τD(exp(−tτT)−exp(−tτD)) 1

where *A* = *N*σ­[*S*_0_]­φ_D_, [^1^O_2_]­(*t*) is the concentration of singlet oxygen as a function of time generated by illumination of *N* photons per cm^2^, σ is the ground-state photosensitizer absorption cross-section at the excitation wavelength, φ_D_ is the photosensitizer singlet-oxygen quantum yield, [*S*_0_] is the photosensitizer concentration, and τ_D_ and τ_T_ are the lifetimes of singlet oxygen and triplet state photosensitizer, respectively. The total number of photons emitted after excitation by a single laser pulse is described as

2 $$\int L_{1270}\left(t\right)dt=\frac{N\sigma \left[S_{0}\right]\varphi_{D}\tau_{D}}{\tau_{R}}$$

here, the detection of ^1^O_2_ generation directly depends on the total emitted luminescence and radiative lifetime of ^1^O_2_ (τ_R_). This approach enabled accurate extraction of ^1^O_2_ lifetimes in vivo, despite the presence of overlapping fast-decay signals from tissue scattering or residual BPD fluorescence.

### 2.4 Validation of TSOLD System for Singlet Oxygen Detection

Before measuring ^1^O_2_ luminescence signature from BPD in mice, validation of the TSOLD system for selective detection of ^1^O_2_ luminescence was performed by measuring the signal using narrow bandpass filters centered at wavelengths of 1200, 1240, 1270, 1300, and 1340 nm (20 nm bandwidth, OD > 6.0, Omega Optical, USA). These filters were individually placed in the detection path between the collection fiber and the SPAD detector, along with the FELH1200 long-pass filter. In vivo validation involved tumor-bearing C3H mice injected with 2 mg/kg BPD. Further validation was performed using a liquid phantom model consisting of 10 mg/kg BPD dissolved in methanol. To verify the specificity of the signal, the same solution was tested with and without the addition of sodium azide (NaN_3_, 20 mM), a chemical quencher of singlet oxygen. The sample was illuminated with a 690 nm laser beam (pulse width 129 ns at 78 kHz repetition rate) with a beam diameter of 1.50 mm and average intensity 130 mW/cm^2^. The signal was acquired with the SPAD operating with 25% detection efficiency and a 13 μs dead time.

### 2.5 Singlet Oxygen Luminescence from BPD in Liquid Phantom

Liquid phantoms (3.5 mL) contained BPD in methanol with 0.6% Intralipid (20% w/v, Fresenius-kabi, USA) (μ_s_ = 8 cm^–1^ at 690 nm) to simulate light scattering in tissue. The BPD concentrations ranged from 1 to 5 mg/kg. Measurements of the ^1^O_2_ luminescence were made in a sealed quartz cuvette (CV10Q35EP, Thorlabs, Newton, NJ, USA) over a period of 5 min. Time histograms comprising 395 bins at 50 ns each were created by tracking single-photon arrivals with the Time Tagger. These histograms were fitted with eq 1 to determine the lifetimes of ^1^O_2_ and the photosensitizer triplet state.

### 2.6 In Vivo BPD Uptake Measurement in Tumour-Bearing Mice

To assess the dynamic uptake of photosensitizer, female C3H mice (6–8 weeks old) bearing subcutaneous RIF tumors (∼3–5 mm diameter) were intravenously injected with BPD (2 mg/kg body weight). ^1^O_2_ measurements were performed in three mice at 5 different postinjection time points: 15 and 30 min, and 1, 2, and 3 h. Before measurements, the tumor region was depilated to minimize optical scattering and autofluorescence. Background signals from control untreated (without BPD) mice were subtracted to isolate the BPD-specific luminescence. This enabled semiquantitative estimation of BPD uptake kinetics in vivo, providing essential context for interpreting the ^1^O_2_ generation at each time point.

### 2.7 Pre and Post-PDT Singlet Oxygen Luminescence Measurements

The same group of C3H mice from the BPD uptake study was used. Each mouse received i.v. injection of BPD (2 mg/kg). PDT was performed 3 h later, delivering the 690 nm treatment light from a 690 nm diode laser (B&W Tek Inc., Newark, DE, USA) through a microlens fiber to produce a collimated beam. The tumor surface irradiance was kept at 150 mW/cm^2^ for 15 min exposure, resulting in a light dose of 135 J/cm^2^. The ^1^O_2_ luminescence was measured immediately before and after PDT.

## 3 Results

Figure 4a shows the ^1^O_2_ luminescence-time histograms for BPD in the liquid phantom, without and following the addition of NaN_3_. Figure 4b shows an example of the in vivo 1270 nm luminescence without and with BPD. The histograms using multiple bandpass filters are shown in Figure 4c.

**Figure 4:**
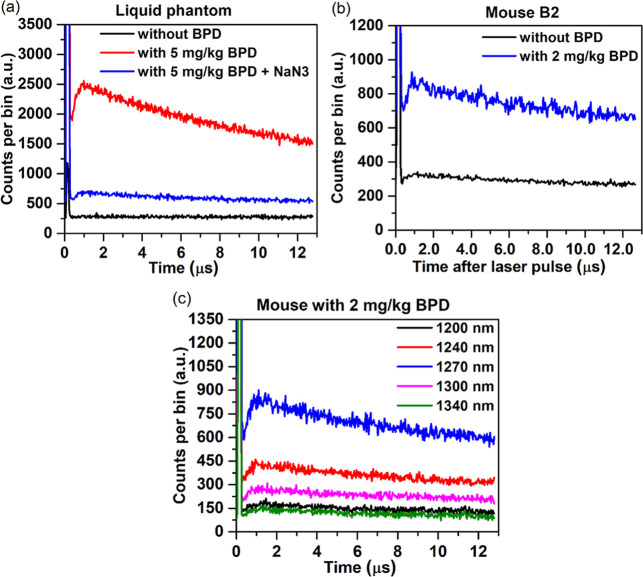
Validation of TSOLD for in vivo singlet oxygen detection. (a) In liquid phantom without BPD, with BPD, and with BPD following addition of NaN_3_, (b) without BPD and with intravenously injected 2 mg/kg BPD in tumor-bearing C3H mice (3 h after BPD injection), confirming photosensitizer-dependent ^1^O_2_ emission. (c) Histograms for 2 mg/kg BPD in mice using discrete band-pass filters.

Loss of the 1270 nm signal following addition of NaN_3_ confirmed that the detected signal originated primarily from ^1^O_2_. The comparison in mice with and without BPD and the spectral data confirms the selective detection of ^1^O_2_ luminescence in vivo, aligning with its known emission signature. These observations validated both the spectral and functional specificity of the TSOLD system for detecting singlet oxygen luminescence under experimental conditions relevant to photodynamic therapy.

Figure 5 presents the time-resolved histograms and cumulative counts for BPD at concentrations ranging from 1 to 5 mg/kg in liquid phantoms, illuminated at 690 nm at an irradiance of 150 mW/cm^2^. The calculated BPD triplet-state lifetime and the ^1^O_2_ lifetime, derived from biexponential fitting of the TSOLD data, along with their corresponding *R* ^2^ and SNR values, are presented in Table 1.

**Figure 5:**
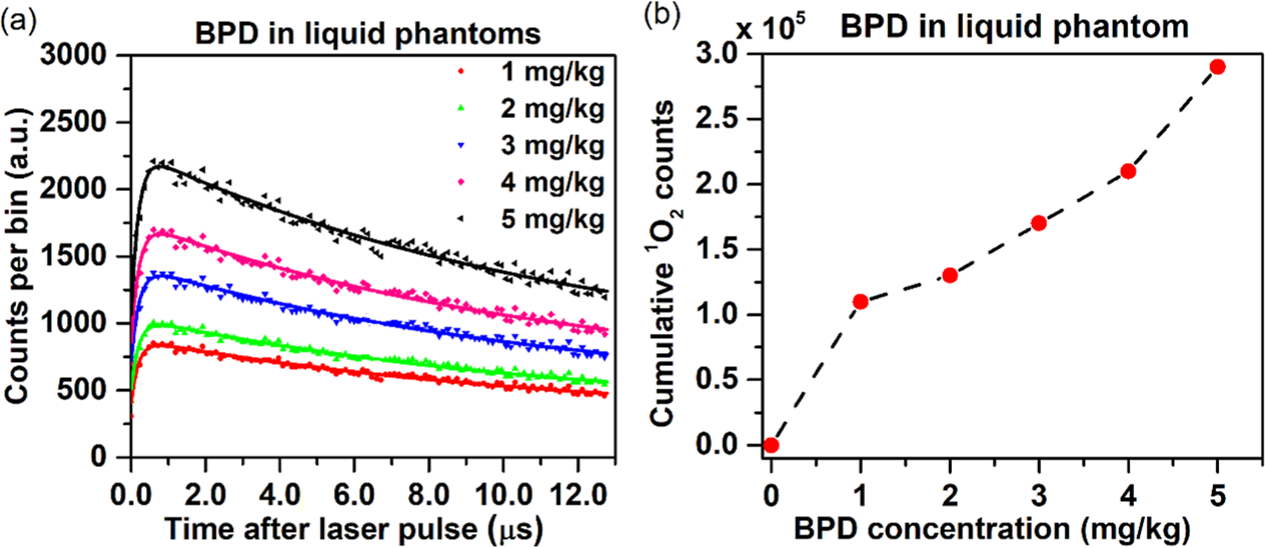
(a) TSOLD single-photon time curves (measured-dots, fitted-lines). (b) BPD concentration-dependent cumulative ^1^O_2_ counts at 1270 nm for BPD concentrations of 1, 2, 3, 4, and 5 mg/kg in liquid phantoms.

**Table 1:** Lifetimes of ^1^O_2_ and BPD Triplet State in Liquid Phantoms, with Corresponding *R* ^2^ and SNR Values from Bi-Exponential Fitting of the TSOLD Data

BPD concentration (mg/kg)	singlet oxygen lifetime (μs)	BPD triplet state lifetime (μs)	coefficient of determination (*R* ^2^)	signal-to-noise ratio
1	11.8 ± 0.1	0.20 ± 0.01	0.97	32.3
2	11.6 ± 0.1	0.21 ± 0.01	0.97	33.7
3	11.5 ± 0.1	0.22 ± 0.01	0.97	34.1
4	11.7 ± 0.1	0.21 ± 0.01	0.97	33.6
5	11.9 ± 0.1	0.20 ± 0.01	0.96	32.8

The luminescence histograms and cumulative counts of ^1^O_2_ demonstrated a linear increase in the number of photons per bin as the concentration of BPD in the liquid phantom increased. As expected, the BPD concentration had minimal impact on the ^1^O_2_ lifetime. The data fit well with eq 1 (*R* ^2^ ∼ 0.97) and the SNR was high (>30). With this validation of the instrument performance, we proceeded to the in vivo studies.

The time histograms and corresponding cumulative ^1^O_2_ photon counts for BPD uptake in 3 mice (B1, B2, B3) at 15, 30, 60, 120, and 180 min postinjection are shown in Figure 6.

**Figure 6:**
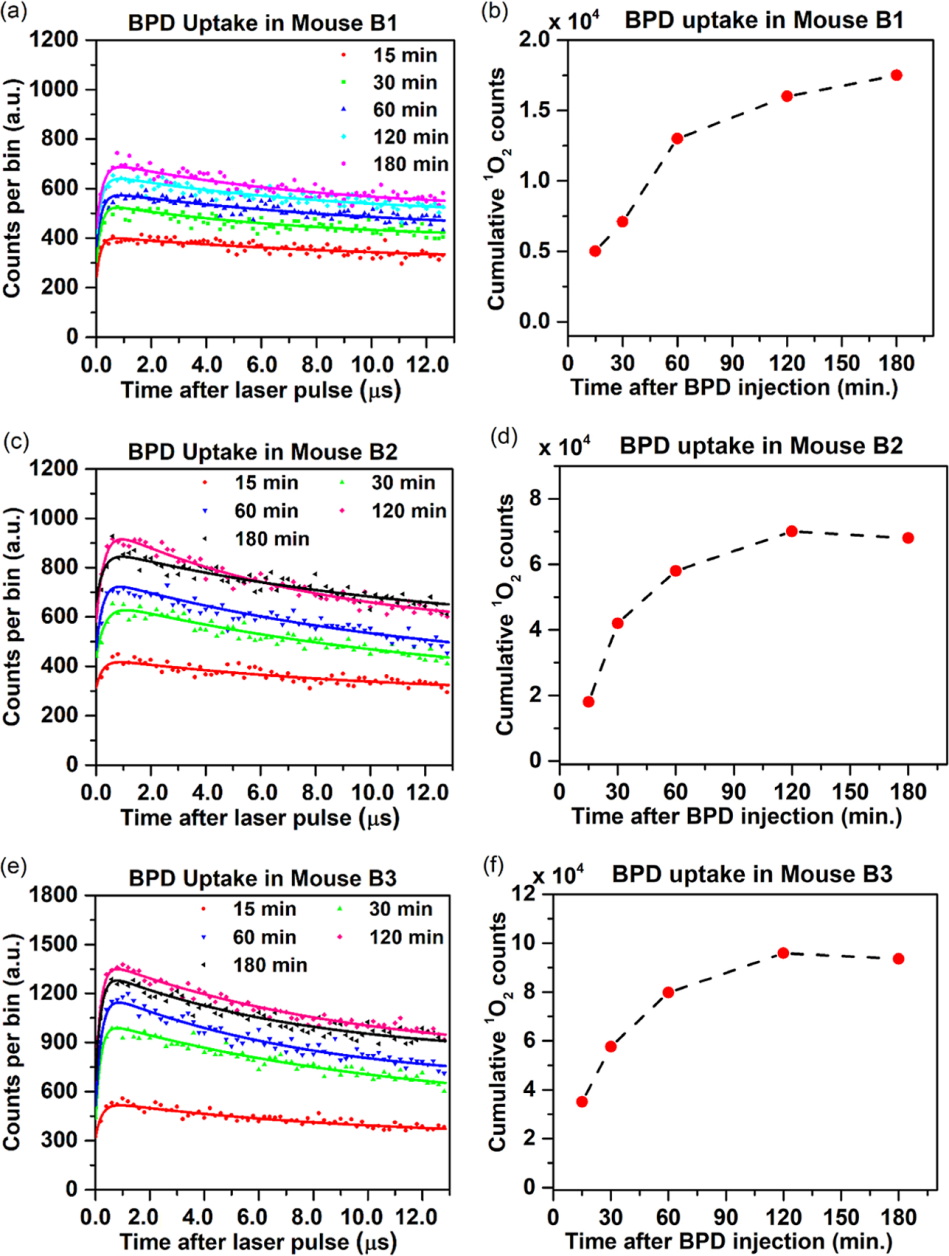
(a,c,e) TSOLD photon counting time curves (measured-dots, fitted-lines) and (b,d,f) cumulative ^1^O_2_ counts in RIF tumors post 2 mg/kg BPD I.V. injection. Acquisition time 5 min.

Figure 6 shows an increase in the BPD counts during the initial uptake phase up to 2 h, with a slight decline by 3 h post injection. The cumulative ^1^O_2_ counts reflect these uptake dynamics, suggesting that ^1^O_2_ generation is directly related to the local concentration of BPD in the tumor. The extracted lifetimes of the BPD triplet state and ^1^O_2_ derived from biexponential curve fitting of the 180 min histogram are given in Table 2.

**Table 2:** Lifetimes of ^1^O_2_ and the BPD Triplet State, with Corresponding *R* ^2^ and SNR Values from Biexponential Fitting of the 180 min Post-Injection TSOLD Data

mouse	singlet oxygen lifetime (μs)	BPD triplet state lifetime (μs)	coefficient of determination (*R* ^2^)	signal-to-noise ratio
B1	0.22 ± 0.1	8.17 ± 1.13	0.62	16.2
B2	0.26 ± 0.1	10.89 ± 0.05	0.82	26.1
B3	0.24 ± 0.04	9.20 ± 0.54	0.93	35.1

The biexponential fitting of the in vivo data matches well with previously reported in vivo lifetime measurements:^3,15,38^ the ^1^O_2_ lifetime ranges from 0.22 to 0.26 μs, which is within the generally reported range of 0.2–4 μs.^3^ The triplet-state lifetimes range from 8.17 to 10.89 μs, which is also within the commonly reported range of 3–40 μs, contingent upon the specific microenvironmental conditions.^3,39,40^ The good *R* ^2^ values (0.80) and favorable SNRs (>15) highlight the reliability of the in vivo measurements.^41^

Following the BPD uptake study, we performed PDT with a CW 690 nm diode laser for 15 min and measured the singlet oxygen luminescence post-PDT, as shown in Figure 7.

**Figure 7:**
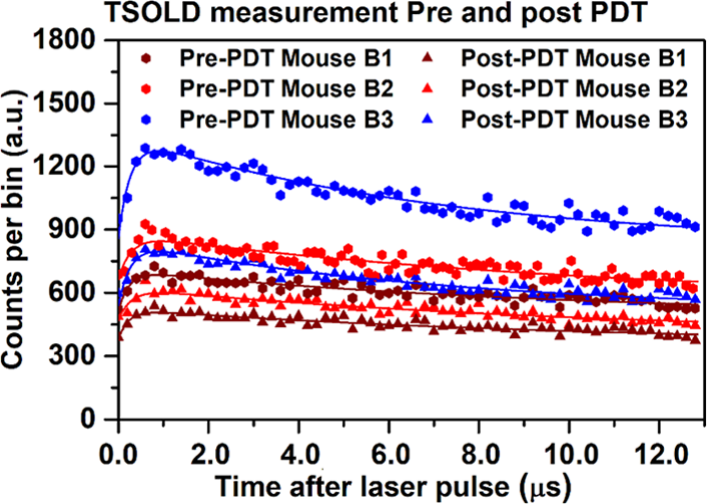
^1^O_2_ photon-counting time curves (measured-dots, fitted-lines) for 3 mouse tumors immediately before and after PDT treatment (2 mg/kg BPD, 3 h drug-light interval, 690 nm, 150 mW/cm^2^ for 15 min).

The post-treatment histograms demonstrated a marked reduction in singlet oxygen counts (by 14, 28, and 37%), indicating significant BPD photobleaching and depletion of available oxygen within the tumor region. These results show the ability of the TSOLD system to track in vivo ^1^O_2_ and photosensitizer concentration that determine the efficacy of the treatment. The consistency of the lifetime estimates with established values further underscores the reliability of the approach.

## 4 Discussion

This study demonstrates the feasibility and reliability of a portable TSOLD instrument using a 690 nm pulsed laser for excitation to quantify both ^1^O_2_ generation and lifetimes and photosensitizer concentration in the target tumor during PDT. The bifurcated fiber bundle allowed simultaneous excitation delivery and ^1^O_2_ luminescence collection at the same site, ensuring spatial coregistration and improving detection efficiency. The choice of 690 nm excitation optimizes the overlap with BPD’s secondary absorption maximum, allowing for deeper tissue penetration while maintaining effective photosensitizer excitation, as reported previously.^42^ The fiber-based light delivery and collection enabled minimally invasive and spatially precise measurements directly at the tumor site, eliminating the need for complex optical alignment. The performance of the system was rigorously validated through a series of controlled experiments. In liquid phantoms containing BPD, robust time-resolved luminescence histograms at 1270 nm were recorded, which were quenched upon adding sodium azide, confirming that the signal originated from ^1^O_2_. In vivo validation involved comparing readings from tumor-bearing mice with and without BPD injection. A clear increase in the 1270 nm signal was observed in the injected mice. Additionally, the time-resolved histograms using bandpass filters at 1200, 1240, 1270, 1300, and 1340 nm showed a distinct peak at 1270 nm in the BPD-injected mice, consistent with typical ^1^O_2_ emission.

The BPD in intralipid-based methanol liquid phantoms showed longer ^1^O_2_ lifetime (∼11.5 μs) compared to the BPD triplet state (∼0.20 μs), which matches previously reported lifetimes for different photosensitizers in methanol phantoms.^3,25^ The high *R* ^2^ (>0.97) and SNR (>30) observed across all BPD concentrations confirm that the TSOLD instrument can reliably resolve ^1^O_2_ and photosensitizer triplet-state decays in a controlled-solvent environment. Also, we examined the residuals from the biexponential fits for all data sets. The residuals show no systematic trends and are randomly distributed around zero, indicating that the fitting model appropriately captures the decay dynamics.

The key finding of this study was the determination of the BPD-mediated PDT treatment response, based on the measured ^1^O_2_ luminescence. The analysis of the postinjection time for BPD, as depicted in Figure 6, demonstrates a significant correlation between BPD uptake in mice (specifically, 2 to 3 h postinjection) and previously reported tumor accumulation times.^43,44^ The slight decline in BPD uptake (or retention) observed in mice B2 and B3 after approximately 2 h, but not in mouse B1, is likely due to variability among the animals in how the photosensitizer is processed and the characteristics of the tumor microenvironment. Factors such as differences in tumor vascularization, blood flow, lymphatic drainage, local oxygen levels, and tissue optical properties can all affect both the delivery and clearance rates of BPD. This variability can lead to differences in retention profiles across the mice, which may influence the detected singlet oxygen signal and its changes over time, especially in in vivo optical measurements. The use of phosphorescence-based methods for detecting BPD uptake shows an efficient method because these techniques do not require a direct assessment of the photochemical activity of the photosensitizer. Both the pre- and post-PDT measurements of ^1^O_2_ luminescence in mice demonstrate that the decrease in ^1^O_2_ luminescence by 20–30% correlated with treatment responses. The reduced ^1^O_2_ may be due to a combination of BPD photobleaching and reduced oxygenation within the tumor microenvironment. BPD is known to be photolabile, which can lead to diminished ^1^O_2_ generation throughout treatment.^45,46^ Additionally, vascular damage and oxygen consumption during PDT can cause temporary hypoxia, further limiting the photochemical generation of ^1^O_2_ and its luminescence.^47,48^ The current results show that TSOLD can detect these photochemical changes in real time, providing feedback for treatment dosimetry and optimization. However, the detection of ^1^O_2_ luminescence is sensitive to submillimeter layers of skin tissue, primarily confined to the epidermis and subdermis, as previously reported,^15,49^ so that fiber-based system may be useful for deeper tissue invasive ^1^O_2_ detection.

## 5 Conclusions

This study demonstrates, for the first time, the development and successful validation of a portable 690 nm excitation wavelength TSOLD instrument for real-time, noninvasive detection of ^1^O_2_ in vivo, with precise estimation of ^1^O_2_ and photosensitizer triplet-state lifetimes. These measurements closely aligned with values documented in the literature, thereby confirming the accuracy and reliability of our system. The in vivo experiments highlight the potential of TSOLD as a sophisticated dosimetry tool for the real-time monitoring of photochemical events during PDT. The specific instrument presented here represents a substantial advance in PDT dose monitoring, enabling optimization of PDT protocols, enhancement of treatment personalization, and the extension of ^1^O_2_ monitoring to a wider array of photosensitizers and clinical applications.

## Data Availability

The data will be made available upon request.

## References

[1] Lowdell C., Ash D., Driver I., Brown S. (1993). Interstitial Photodynamic Therapy.
Clinical Experience with Diffusing Fibres in the Treatment of Cutaneous
and Subcutaneous Tumours. Br. J. Cancer.

[2] Fisher A. M. R., Murphree A. L., Gomer C. J. (1995). Clinical
and Preclinical Photodynamic
Therapy. Lasers Surg. Med..

[3] Niedre M., Patterson M. S., Wilson B. C. (2002). Direct Near-Infrared
Luminescence
Detection of Singlet Oxygen Generated by Photodynamic Therapy in Cells
In Vitro and Tissues In Vivo. Photochem. Photobiol..

[4] Hackbarth S., Islam W., Fang J., Subr V., Röder B., Etrych T., Maeda H. (2019). Singlet Oxygen
Phosphorescence Detection
in Vivo Identifies PDT-Induced Anoxia in Solid Tumors. Photochem. Photobiol. Sci..

[5] Henderson B. W., Dougherty T. J. (1992). HOW DOES PHOTODYNAMIC THERAPY WORK?. Photochem. Photobiol..

[6] Epe B., Pflaum M., Boiteux S. (1993). DNA Damage
Induced by Photosensitizers
in Cellular and Cell-Free Systems. Mutat. Res.
Genet. Toxicol..

[7] Epe B. (2012). DNA Damage
Spectra Induced by Photosensitization. Photochem.
Photobiol. Sci..

[8] Agostinis P., Berg K., Cengel K. A., Foster T. H., Girotti A. W., Gollnick S. O., Hahn S. M., Hamblin M. R., Juzeniene A., Kessel D., Korbelik M., Moan J., Mroz P., Nowis D., Piette J., Wilson B. C., Golab J. (2011). Photodynamic
Therapy of Cancer: An Update. Ca-Cancer J. Clin..

[9] El-Hussein A., Harith M., Abrahamse H. (2012). Assessment
of DNA Damage after Photodynamic
Therapy Using a Metallophthalocyanine Photosensitizer. Int. J. Photoenergy.

[10] Moghissi K., Dixon K., Thorpe J. A. C., Stringer M., Moore P. J. (2000). The Role
of Photodynamic Therapy (PDT) in Inoperable Oesophageal Cancer. Eur. J. Cardio. Thorac. Surg..

[11] Shafirstein G., Battoo A., Harris K., Baumann H., Gollnick S. O., Lindenmann J., Nwogu C. E. (2016). Photodynamic Therapy
of Non–Small
Cell Lung Cancer. Narrative Review and Future Directions. Ann. Am. Thorac. Soc..

[12] Kazemi K. S., Kazemi P., Mivehchi H., Nasiri K., Eshagh Hoseini S. S., Nejati S. T., Pour
Bahrami P., Golestani S., Nabi Afjadi M. (2024). Photodynamic
Therapy: A Novel Approach for Head and
Neck Cancer Treatment with Focusing on Oral Cavity. Biol. Proced. Online.

[13] Jarvi M. T., Niedre M. J., Patterson M. S., Wilson B. C. (2006). Singlet Oxygen Luminescence
Dosimetry (SOLD) for Photodynamic Therapy: Current Status, Challenges
and Future Prospects. Photochem. Photobiol..

[14] Niedre M. J., Patterson M. S., Giles A., Wilson B. C. (2005). Imaging of Photodynamically
Generated Singlet Oxygen Luminescence In Vivo. Photochem. Photobiol..

[15] Niedre M. J., Yu C. S., Patterson M. S., Wilson B. C. (2005). Singlet Oxygen Luminescence
as an in Vivo Photodynamic Therapy Dose Metric: Validation in Normal
Mouse Skin with Topical Amino-Levulinic Acid. Br. J. Cancer.

[16] Yang W., Johnson M., Lu B., Sourvanos D., Sun H., Dimofte A., Vikas V., Busch T. M., Hadfield R. H., Wilson B. C., Zhu T. C. (2024). Correction of Multispectral Singlet
Oxygen Luminescent Dosimetry (MSOLD) for Tissue Optical Properties
in Photofrin-Mediated Photodynamic Therapy. Antioxidants.

[17] Scholz M., Dědic R., Valenta J., Breitenbach T., Hála J. (2014). Real-Time
Luminescence Microspectroscopy Monitoring
of Singlet Oxygen in Individual Cells. Photochem.
Photobiol. Sci..

[18] Lu, B. ; Yang, W. ; Sun, H. ; Johnson, M. ; Vikas, V. ; Cherop, F. ; Wilson, B. C. ; Hadfield, R. ; Zhu, T. C. Adanced Photodynamic Therapy Monitoring Using a 7-Channel Continuous Singlet Oxygen Luminescent Dosimetry (CSOLD) Device. In Optical Methods for Tumor Treatment and Detection: Mechanisms and Techniques in Photodynamic Therapy XXXIII, 2013; p 28.

[19] Yang, W. ; Lu, B. ; Vikas, V. ; Johnson, M. ; Sun, H. ; Wilson, B. C. ; Hadfield, R. H. ; Zhu, T. C. Comparative Analysis of Multichannel Singlet Oxygen Luminescence Dosimetry (MSOLD) and Singlet Oxygen Explicit Dosimetry (SOED) in Benzoporphyrin Derivative (BPD)-Mediated Photodynamic Therapy: A Mouse Model Study. In Optical Methods for Tumor Treatment and Detection: Mechanisms and Techniques in Photodynamic Therapy XXXIII, 2013; p 27.

[20] Gemmell N. R., McCarthy A., Liu B., Tanner M. G., Dorenbos S. D., Zwiller V., Patterson M. S., Buller G. S., Wilson B. C., Hadfield R. H. (2013). Singlet Oxygen Luminescence
Detection with a Fiber-Coupled
Superconducting Nanowire Single-Photon Detector. Opt. Express.

[21] Gemmell N. R., McCarthy A., Kim M. M., Veilleux I., Zhu T. C., Buller G. S., Wilson B. C., Hadfield R. H. (2017). A Compact Fiber-optic
Probe-based Singlet Oxygen Luminescence Detection System. J. Biophot..

[22] Kim M., Penjweini R., Gemmell N., Veilleux I., McCarthy A., Buller G., Hadfield R., Wilson B., Zhu T. (2016). A Comparison
of Singlet Oxygen Explicit Dosimetry (SOED) and Singlet Oxygen Luminescence
Dosimetry (SOLD) for Photofrin-Mediated Photodynamic Therapy. Cancers.

[23] Sykes A., Saalbach L., Benson S., Nestoros E., Tobin R., Yi X., Tanner M. G., Vendrell M., Buller G. S. (2025). Real-Time Detection
of Singlet-Oxygen Signatures Using a Single-Photon Avalanche Diode
Detector. Biomed. Opt. Express.

[24] Vikas, V. ; Lu, B. ; Yang, W. ; Wilson, B. C. ; Zhu, T. C. ; Hadfield, R. H. Impact of Scattering Medium on Singlet Oxygen Luminescence Generation in Photodynamic Therapy. In Optical Methods for Tumor Treatment and Detection: Mechanisms and Techniques in Photodynamic Therapy XXXIII, 2013; p 21.

[25] Vikas V., Yang W., Wilson B. C., Zhu T. C., Hadfield R. H. (2025). Analysis
of Singlet Oxygen Luminescence Generated By Protoporphyrin IX. Antioxidants.

[26] Tsimvrakidis K., Gemmell N. R., Erotokritou K., Miki S., Yabuno M., Yamashita T., Terai H., Hadfield R. H. (2019). Enhanced Optics
for Time-Resolved Singlet Oxygen Luminescence Detection. IEEE J. Select. Topics Quantum Electron..

[27] Boso G., Ke D., Korzh B., Bouilloux J., Lange N., Zbinden H. (2016). Time-Resolved
Singlet-Oxygen Luminescence Detection with an Efficient and Practical
Semiconductor Single-Photon Detector. Biomed.
Opt. Express.

[28] Hernández I. C., Buttafava M., Boso G., Diaspro A., Tosi A., Vicidomini G. (2015). Gated STED
Microscopy with Time-Gated Single-Photon
Avalanche Diode. Biomed. Opt. Express.

[29] Richter A. M., Cerruti-Sola S., Sternberg E. D., Dolphin D., Levy J. G. (1990). Biodistribution
of Tritiated Benzoporphyrin Derivative (3H-BPD-MA), a New Potent Photosensitizer,
in Normal and Tumor-Bearing Mice. J. Photochem.
Photobiol., B.

[30] Schmidt-Erfurth U., Hasan T., Gragoudas E., Michaud N., Flotte T. J., Birngruber R. (1994). Vascular Targeting
in Photodynamic Occlusion of Subretinal
Vessels. Ophthalmology.

[31] Akens M. K., Hardisty M. R., Wilson B. C., Schwock J., Whyne C. M., Burch S., Yee A. J. M. (2010). Defining
the Therapeutic Window of
Vertebral Photodynamic Therapy in a Murine Pre-Clinical Model of Breast
Cancer Metastasis Using the Photosensitizer BPD-MA (Verteporfin). Breast Cancer Res. Treat..

[32] Baglo Y., Sorrin A. J., Liang B. J., Huang H. (2020). Harnessing
the Potential
Synergistic Interplay Between Photosensitizer Dark Toxicity and Chemotherapy. Photochem. Photobiol..

[33] Glidden M. D., Celli J. P., Massodi I., Rizvi I., Pogue B. W., Hasan T. (2012). Image-Based Quantification of Benzoporphyrin Derivative Uptake, Localization,
and Photobleaching in 3D Tumor Models, for Optimization of PDT Parameters. Theranostics.

[34] Cui S., Guo X., Wang S., Wei Z., Huang D., Zhang X., Zhu T. C., Huang Z. (2024). Singlet Oxygen in Photodynamic Therapy. Pharmaceuticals.

[35] Kim M. M., Penjweini R., Liang X., Zhu T. C. (2016). Explicit Macroscopic
Singlet Oxygen Modeling for Benzoporphyrin Derivative Monoacid Ring
A (BPD)-Mediated Photodynamic Therapy. J. Photochem.
Photobiol., B.

[36] Kim M. M., Penjweini R., Zhu T. C. (2017). Evaluation of Singlet Oxygen Explicit
Dosimetry for Predicting Treatment Outcomes of Benzoporphyrin Derivative
Monoacid Ring A-Mediated Photodynamic Therapy. J. Biomed. Opt..

[37] Zhu T. C., Kim M. M., Liang X., Finlay J. C., Busch T. M. (2015). In-Vivo
Singlet Oxygen Threshold Doses for PDT. Photon.
Laser Med..

[38] Baker A., Kanofsky J. R. (1992). QUENCHING OF SINGLET
OXYGEN BY BIOMOLECULES FROM L1210
LEUKEMIA CELLS. Photochem. Photobiol..

[39] Looft A., Pfitzner M., Preuß A., Röder B. (2018). In Vivo Singlet
Molecular Oxygen Measurements: Sensitive to Changes in Oxygen Saturation
during PDT. Photodiagnosis Photodyn. Ther..

[40] Hackbarth S., Islam R., Šubr V., Etrych T., Fang J. (2022). Singlet Oxygen
In Vivo: It Is All about Intensity. JPM.

[41] Chicco D., Warrens M. J., Jurman G. (2021). The Coefficient of
Determination
R-Squared Is More Informative than SMAPE, MAE, MAPE, MSE and RMSE
in Regression Analysis Evaluation. PeerJ Comput.
Sci..

[42] Ash C., Dubec M., Donne K., Bashford T. (2017). Effect of Wavelength
and Beam Width on Penetration in Light-Tissue Interaction Using Computational
Methods. Lasers Med. Sci..

[43] Chen B., Pogue B. W., Hasan T. (2005). Liposomal
Delivery of Photosensitising
Agents. Expet Opin. Drug Deliv..

[44] Allison R. R., Downie G. H., Cuenca R., Hu X.-H., Childs C. J., Sibata C. H. (2004). Photosensitizers
in Clinical PDT. Photodiagnosis Photodyn. Ther..

[45] Stratonnikov, A. A. ; Meerovich, G. A. ; Loschenov, V. B. In Photobleaching of Photosensitizers Applied for Photodynamic Therapy; Dougherty, T. J. , Ed.; SPIE: San Jose, CA, 2000; pp 81–91.

[46] Johansson A., Faber F., Kniebühler G., Stepp H., Sroka R., Egensperger R., Beyer W., Kreth F. (2013). Protoporphyrin IX Fluorescence
and Photobleaching During Interstitial Photodynamic Therapy of Malignant
Gliomas for Early Treatment Prognosis. Lasers
Surg. Med..

[47] Shen Z., Ma Q., Zhou X., Zhang G., Hao G., Sun Y., Cao J. (2021). Strategies
to Improve Photodynamic Therapy Efficacy by Relieving
the Tumor Hypoxia Environment. NPG Asia Mater..

[48] Karwicka M., Pucelik B., Gonet M., Elas M., Dąbrowski J. M. (2019). Effects
of Photodynamic Therapy with Redaporfin on Tumor Oxygenation and Blood
Flow in a Lung Cancer Mouse Model. Sci. Rep..

[49] Robinson D. J., De Bruijn H. S., Van Der Veen N., Stringer M. R., Brown S. B., Star W. M. (1998). Fluorescence
Photobleaching of ALA-induced Protoporphyrin
IX during Photodynamic Therapy of Normal Hairless Mouse Skin: The
Effect of Light Dose and Irradiance and the Resulting Biological Effect. Photochem. Photobiol..

